# Quantitative Angiographic Assessment of Aortic Regurgitation Following 11 TAVR Devices: An Update of a Multicenter Pooled Analysis

**DOI:** 10.1016/j.jscai.2022.100037

**Published:** 2022-04-14

**Authors:** Mahmoud Abdelshafy, Patrick W. Serruys, Won-Keun Kim, Andreas Rück, Rutao Wang, Ling Tao, Ahmed Elkoumy, Hesham Elzomor, Scot Garg, Yoshinobu Onuma, Darren Mylotte, Osama Soliman

**Affiliations:** a Department of Cardiology, National University of Ireland, Galway (NUIG), and CORRIB Research Centre for Advanced Imaging and Core Laboratory, Galway, Ireland; b Department of Cardiology, Al-Azhar University, Cairo, Egypt; c NHLI, Imperial College London, London, United Kingdom; d CÚRAM, SFI Research Centre for Medical Devices, Galway, Ireland; e Department of Cardiology, Kerckhoff Heart Centre, Bad Nauheim, Germany; f Department of Cardiology, Karolinska Institute, Stockholm, Sweden; g Department of Cardiology, Xijing Hospital, Xi'an, China; h Islamic Center of Cardiology and Cardiac Surgery, Al-Azhar University, Cairo, Egypt; i Department of Cardiology, Royal Blackburn Hospital, Blackburn, United Kingdom

**Keywords:** Transcatheter aortic valve replacement, paravalvular leak, aortic regurgitation, videodensitometry, transcatheter aortic valve implantation

## Abstract

**Background:**

Aortic regurgitation (AR) following transcatheter aortic valve replacement (TAVR) is a major predictor of short- and long-term survival. Thus far, no independent quantitative comparison of AR among commercially available transcatheter heart valves (THVs) has been performed.

**Objectives:**

We sought to assess and compare the degree of acute AR following TAVR between 11 commercially available THVs and update our previous multicenter, pooled analysis.

**Methods:**

Analyses were performed by an independent academic core lab using quantitative videodensitometry, a technique relying solely on the aortogram acquired after TAVR. The pooled analysis (*n* = 2665) included data from the initial cohort of 7 valves (Lotus [*n* = 546], Evolut PRO [*n* = 95], SAPIEN 3 [*n* = 397], Evolut R [*n* = 295], SAPIEN XT [*n* = 239], ACURATE neo [*n* = 120], and CoreValve [*n* = 532]) to which data from 4 new valves were added (ACURATE neo2 [*n* = 120], Myval [*n* = 108], VitaFlow [*n* = 105], and Venus-A [*n* = 113]).

**Results:**

The Lotus valve had the lowest mean AR (3.5% ± 4.4%) followed by ACURATE neo2 (4.4% ± 4.8%), VitaFlow (6.1% ± 6.4%), Myval (6.3% ​± ​6.3%), Evolut PRO (7.4% ± 6.5%), SAPIEN 3 (7.6% ± 7.1%), Evolut R (7.9% ± 7.4%), SAPIEN XT (8.8% ± 7.5%), Venus-A (8.9% ​± ​10%), ACURATE neo (9.6% ± 9.2%), and CoreValve (13.7% ± 10.7%, analysis of variance *P*-value < .001). The only valves that statistically differed from all their counterparts were Lotus, with the lowest regurgitation in comparison to other valves except ACURATE neo2, which had less regurgitation compared with SAPIEN 3, Evolut R, SAPIEN XT, Venus-A, ACURATE neo, and CoreValve. CoreValve had the highest mean of AR, with the rates of moderate/severe AR: ACURATE neo2 (1.7%), Lotus (2.2%), Myval (2.8%), VitaFlow (4.7%), Evolut PRO (5.3%), SAPIEN 3 (8.3%), Evolut R (8.8%), SAPIEN XT (10.9%), ACURATE neo (11.3%), Venus-A (14.2%), and CoreValve (30.1%)—χ^2^*P*-value < .001.

**Conclusions:**

In this updated pooled analysis, the Lotus valve had the lowest mean AR, while ACURATE neo2 had the lowest rate of moderate/severe AR. Myval, VitaFlow, and Venus-A THVs showed promising results.

## Highlights

• Aortic regurgitation following transcatheter aortic valve replacement (TAVR) negatively affects patient outcomes.
• We compared independently aortic regurgitation immediately after TAVR between 11 devices.
• Analysis was done using videodensitometry, which is an objective and validated tool.
• The new sealing features effectively reduce the incidence of significant paravalvular regurgitation (PVR).
• Incidence of mild PVR is still prominent with all TAVR devices, requiring more curiosity.

## Central Illustration

**Figure:**
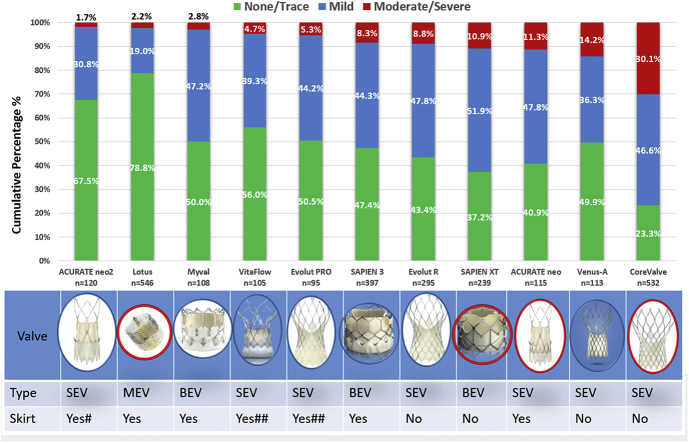

## Teaser

An independent quantitative assessment of regurgitation following TAVR using 11 different devices was performed in 2665 patients and showed that the mean rate of regurgitation was lowest with the Lotus valve (3.5% ± 4.4%) followed by ACURATE neo2 (4.4% ± 4.8%), VitaFlow (6.1% ± 6.4%), Myval (6.3% ​± ​6.3%), Evolut PRO (7.4% ± 6.5%), SAPIEN 3 (7.6% ± 7.1%), Evolut R (7.9% ± 7.4%), SAPIEN XT (8.8% ± 7.5%), Venus-A (8.9% ​± ​10%), ACURATE neo (9.6% ± 9.2%), and CoreValve (13.7% ± 10.7%, ANOVA *P*-value < .001). The only valves that differed statistically from their counterparts were Lotus, with the lowest regurgitation in comparison to other valves except ACURATE neo2, which had less regurgitation compared with SAPIEN 3, Evolut R, SAPIEN XT, Venus-A, ACURATE neo, and CoreValve. CoreValve had the highest mean of regurgitation compared to all other valves.

## Introduction

Moderate or severe aortic regurgitation (AR) after transcatheter aortic valve replacement (TAVR) impacts all-cause mortality,^1^^,^^2^ and thus, as TAVR indications expand into younger patients with longer life expectancy, there is an increasing need to accurately assess it after implantation. In this regard, quantitative videodensitometric assessment of AR has been extensively vetted and validated in vitro,^3^^,^^4^ in vivo,^5^ and in the clinical setting, such as after TAVR.^6^, ^7^, ^8^, ^9^, ^10^, ^11^, ^12^, ^13^, ^14^, ^15^, ^16^, ^17^, ^18^, ^19^ Notably, this technique is accepted as an objective, accurate, and reproducible tool that has been advocated, among other techniques, in the Valve Academic Research Consortium 3 consensus as a reliable modality for assessing AR.^20^

Randomized clinical trials comparing TAVR devices in a head-to-head fashion have been widely used to investigate novel transcatheter heart valve (THV) systems, with 6 randomized controlled trials published between 2014 and 2020,^21^, ^22^, ^23^, ^24^, ^25^, ^26^ although they have only compared a maximum of 2 to 3 devices. Previously we published a multicenter-pooled analysis comparing immediate AR after implantation of 7 different TAVR devices using quantitative angiographic assessment and found that the least amount of regurgitation was seen with the Lotus valve.^10^ With the rapid expansion of the TAVR market and the introduction of many new THV systems, we aimed to update our previous analysis to include 4 new valves.

## Methods

### Study population

The present study is a retrospective analysis of aortograms from a multicenter, multicontinental cohort of consecutive patients who underwent TAVR according to each participating institution's heart team recommendation. The previous cohort analysis included data from 7 valves: Lotus (*n* = 546), Evolut PRO (*n* = 95), SAPIEN 3 (*n* = 397), Evolut R (*n* = 295), SAPIEN XT (*n* = 239), ACURATE neo (*n* = 120), and CoreValve (*n* = 532).^10^ This updated pooled analysis includes additional data from 446 aortograms performed following implantation of 4 new valves: ACURATE neo2 (Boston Scientific) (*n* = 120),^27^ Myval (Meril Life Sciences Pvt. Ltd) (*n* = 108),^7^ VitaFlow (MicroPort) (*n* = 105), and Venus-A (Venus Medtech Inc) (*n* = 113).^28^ The only inclusion criterion in these series was the consecutive acquisition of aortograms. The study complied with the Declaration of Helsinki and Good Clinical Practice guidelines and was approved by each institution's ethics committees. A list of collaborating centers, investigators, recruitment period, and operator experience is provided in Supplemental Table S1 for the initial cohort of 7 THVs and Supplemental Table S2 for the 4 newly added THVs.

### Angiographic quantitative assessment of regurgitation

Analysis was done by quantitative videodensitometric aortography (left ventricular outflow tract [LVOT]-AR) as previously described.^10^ This technique relies solely on the aortogram acquired after TAVR. Briefly, the software (CAAS A-valve, Pie Medical Imaging) constructs 2 time-density curves assessed in 2 regions of interest: the aortic root where the contrast is injected and the LVOT where the regurgitation is quantified. The ratio between the areas under the 2 time-density curves of these regions is the AR in percentage (Supplementary Video 1). The analysis was performed by an independent academic core laboratory not financially subsidized by the industry.

### Statistical analysis

Continuous variables were reported as mean ​± ​standard deviations. Comparison of LVOT-AR was performed using one-way analysis of variance and 2-by-2 comparisons using the post hoc Bonferroni test. Stratification of continuous variable regurgitation into categorical variables was performed according to the following predetermined threshold criteria: (1) none/trace regurgitation (LVOT-AR < 6%), (2) mild (6% ≤ LVOT-AR ≤ 17%), and (3) moderate/severe (LVOT-AR > 17%). The proportion of patients with moderate or severe AR (LVOT-AR > 17%) was compared using a χ^2^ test. A 2-sided *P*-value of .05 was considered indicative of statistical significance. Statistical analyses were performed with SPSS, version 25.0 (IBM).

## Results

Out of the 4587 post-TAVR aortograms, 2665 (58.1%) were suitable for analysis by quantitative assessment of AR, with the following main reasons for which aortograms could not be analyzed: overlapping of the descending aorta with the LVOT (region of interest), overlapping of the descending aorta on the ascending aorta, deep breathing, or table motion. The analyzable aortograms included the following valves: Lotus (*n* ​= ​546), Evolut PRO (*n* = 95), SAPIEN 3 (*n* = 397), Evolut R (*n* = 295), SAPIEN XT (*n* = 239), ACURATE neo (*n* = 120), CoreValve (*n* = 532), ACURATE neo2 (*n* = 120), Myval (*n* = 108), VitaFlow (*n* = 105), and Venus-A (*n* = 113).

**Figure 1:**
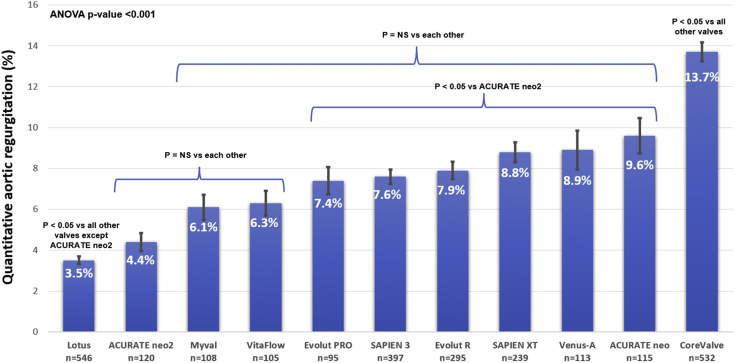
Comparison of the mean LVOT-AR after TAVR among the 11 THVs. Bars denote the mean regurgitation values, and error bars denote standard errors of the mean. ANOVA, analysis of variance; AR, aortic valve regurgitation; LVOT-AR, quantitative aortic regurgitation in the left ventricular outflow tract; NS, non-significant *P*-value; TAVR, transcatheter aortic valve replacement; THV, transcatheter heart valve.

Among the valves included in the pooled analysis, the Lotus valve had the lowest mean AR (3.5% ± 4.4%) followed by ACURATE neo2 (4.4% ± 4.8%), VitaFlow (6.1% ± 6.4%), Myval (6.3% ​± ​6.3%), Evolut PRO (7.4% ± 6.5%), SAPIEN 3 (7.6% ± 7.1%), Evolut R (7.9% ± 7.4%), SAPIEN XT (8.8% ± 7.5%), Venus-A (8.9% ​± ​10%), ACURATE neo (9.6% ± 9.2%), and CoreValve (13.7% ± 10.7%, analysis of variance *P*-value < .001 Figure 1). Post hoc 2-by-2 testing revealed that Lotus had significantly (*P* ​< ​.05) lower regurgitation than each of the other valves except for ACURATE neo2, which had significantly (*P* ​< ​.05) lower regurgitation than SAPIEN 3, Evolut R, SAPIEN XT, Venus-A, ACURATE neo, and CoreValve. Similarly, the first-generation CoreValve had significantly higher regurgitation than each of the other valves. Apart from CoreValve, Lotus, and ACURATE neo2, no other valve differed in the amount of regurgitation from each other.

### Moderate or severe regurgitation

The proportion of patients with moderate or severe regurgitation, within each THV group, followed the same ranking as seen when valve regurgitation was a continuous variable. The ACURATE neo2 valve had the lowest proportion of patients with an LVOT-AR >17% (1.7%), followed by the Lotus (2.2%), Myval (2.8%), VitaFlow (4.7%), Evolut PRO (5.3%), SAPIEN 3 (8.3%), Evolut R (8.8%), SAPIEN XT (10.9%), ACURATE neo (11.3%), Venus-A (14.2%), and CoreValve (30.1%)—χ^2^ *P*-value < .001 (Central Illustration).

**Central Illustration:**
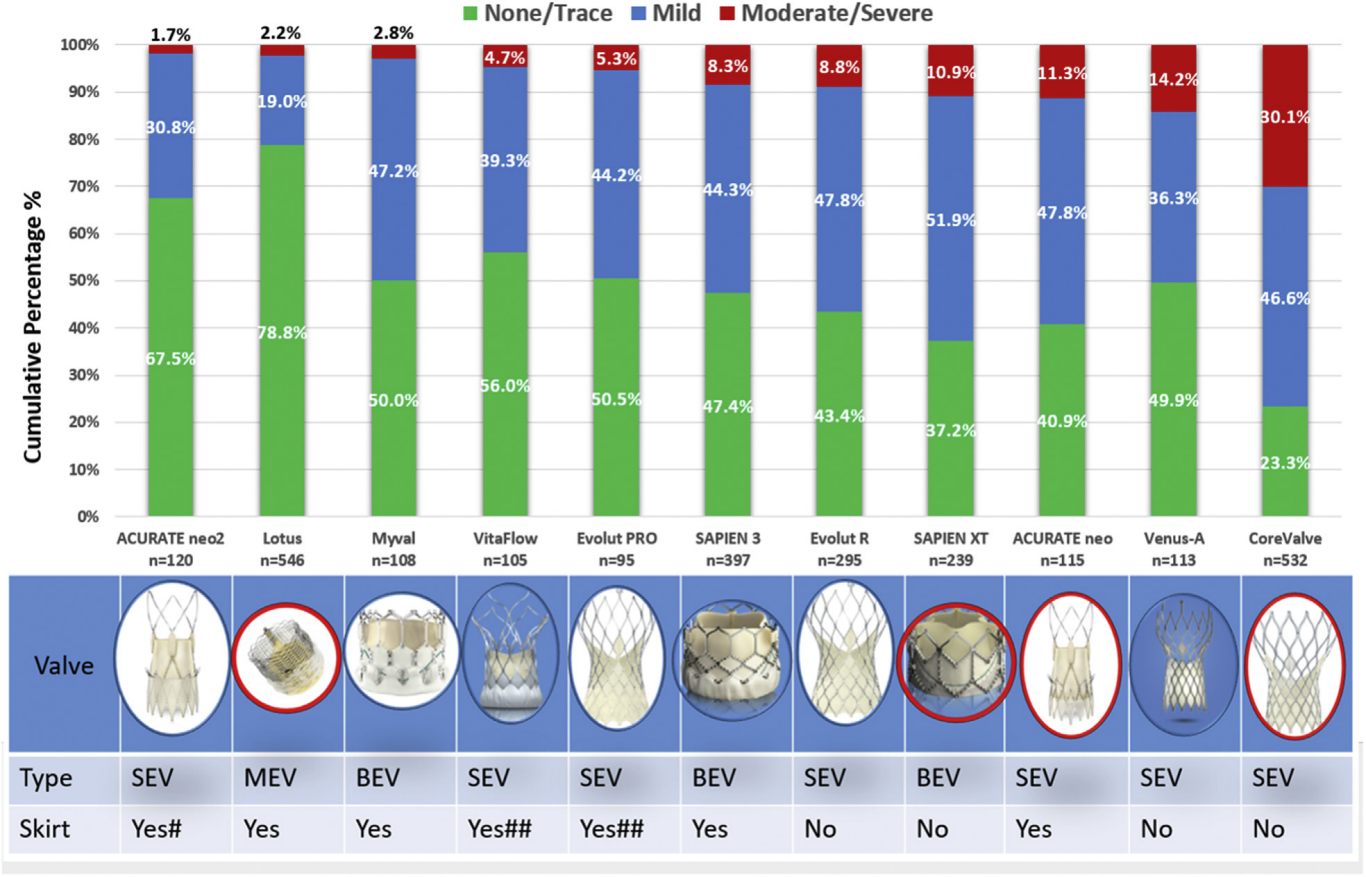
The upper panel shows the cumulative percentage of different degrees of post-TAVR AR by videodensitometric assessment. The lower panel shows photo, type, and presence of the antileak skirt. AR, aortic valve regurgitation; BEV, balloon-expandable valve; MEV, mechanically expandable valve; SEV, self-expanding valve; TAVR, transcatheter aortic valve replacement. # A higher outer skirt compared to ACURATE neo, ## antileak skirt ​+ ​pericardial wrap, and devices circled in red are not commercially available anymore.

On the one hand, in the first-in-man study of the VitaFlow THV, 8 patients required a valve-in-valve procedure due to inappropriate positioning of the valve resulting in severe paravalvular regurgitation. On the other hand, a landmark analysis comparing the first 56 patients treated with the Venus A-valve to the next 57 patients revealed a significantly lower mean LVOT-AR in the later cohort (11.3% ​± ​11.9% vs 6.5% ​± ​7.1%, *P* ​= ​.011).

## Discussion

The main findings of the present study are as follows:

I. The addition of anti–paravalvular regurgitation (PVR) sealing features to new-generation THVs proved to be effective in reducing the incidence of significant PVR in comparison with first-generation THVs.
II. The newly added THVs (ACURATE neo2, Myval, VitaFlow, and Venus A) had comparable results to the THVs analyzed in our previous pooled analysis.
III. Of note, although the incidence of moderate/severe AR is less common with new generations of THVs, the incidence of mild AR is still prominent with all THVs: mild AR % around 30% to 50% except with the Lotus valve 19%.

To the best of our knowledge, this is the first head-to-head objective comparison of 11 different THVs in a large cohort of consecutive “real-world” patients, conducted by the same independent core lab.

The severity of PVR depends on the interaction between anatomical characteristics of the native aortic valve (bicuspid leaflet, elliptical annulus, calcified cups, etc.), the THV platform, and the implantation technique. Modifications in the design of THVs, such as radial force, sealing skirts, frame composition, or size of struts, may, among others, influence the THV's sealing capacity.^10^

Videodensitometric analysis of regurgitation is an objective method of evaluating regurgitation derived from time-density analysis of the angiogram. It has been validated in silico with a mock circulation and correlates strongly with the actual regurgitation flow measured using a flow probe.^4^ Moreover, it has been validated in vivo in the porcine model.^5^ In the clinical setting, previous studies have demonstrated that an LVOT-AR > 17% corresponds to moderate or severe AR in comparison with transthoracic or transesophageal echocardiogram and was associated with an increase in mortality after TAVR.^13^^,^^17^ Likewise, videodensitometric assessment of PVR severity after TAVR correlates well with the cardiac magnetic resonance–derived regurgitation fraction (CMR-RF). The average CMR-RF was 6.7% ± 7.0%, whereas the average LVOT-AR was 7.0% ± 7.0%, with a substantial correlation (r = 0.78, *P* < .001).^16^ In addition, videodensitometric analysis has been shown to be highly reproducible^6^^,^^16^^,^^18^ with, for example, respective interobserver and intraobserver correlation coefficients of 0.95 (*P* ​< ​.0001; Bland-Altman mean difference 0.01 ​± ​0.04; *P* ​= ​.3261) and 0.97 (*P* ​< ​.0001; Bland-Altman mean difference 0.01 ​± ​0.05; *P* ​= ​.5280) in the Brazilian registry cohort.^18^ In the recently published Valve Academic Research Consortium-3 consensus,^29^ AR by videodensitometry is acknowledged as a valid quantitative assessment, although Doppler echocardiography remains by tradition and convention the primary modality for assessing and comparing regurgitation after TAVR.

The introduction of the antileak skirt appears to have greatly reduced AR with THVs, as shown in our analysis by the improvement in post-TAVR PVR following introduction and improvement in the skirt. Quantitative aortographic analysis reveals a 12.2% absolute risk reduction in the rate of moderate or severe AR with the ACURATE neo2 when compared with the ACURATE neo THV. The newly designed ACURATE neo2 is equipped with a 60% larger inner and outer skirt made of a specific material to comply with the calcified and irregular annulus anatomy in the device landing zone. The ACURATE neo2 is also equipped with radiopaque positioning markers for accurate placement, which may have mitigated the severity of PVR with the neo2 iteration.^27^ Additionally, our pooled analysis shows promising results with balloon-expandable valves (BEVs) and self-expandable valves (SEVs) developed in Asia. In comparison with the other BEVs analyzed in our database, the Indian BEV Myval had the lowest rate of moderate or severe AR after TAVR, which is in part due to its unique additional intermediate and extra-large sizes (20 ​mm, 21.5 ​mm, 23 ​mm, 24.5 ​mm, 26 ​mm, 27.5 ​mm, 29 ​mm, 30.5 ​mm, and 32 ​mm) helping eliminate the risk of under-sizing or oversizing, along with the special design of its internal and external skirts.^7^^,^^30^ The outstanding performance of the Myval THV in comparison to the other available BEVs is in line with the result of other studies.^31^^,^^32^ Likewise, the Chinese valves, VitaFlow and Venus-A, have acceptable results even in Chinese patients with their more challenging anatomy such as high calcification and bicuspid anatomy.^28^ VitaFlow and Venus-A are SEVs and have received China Food and Drug Administration approval and are used in daily clinical practice in China. The learning curves shown with these 2 THVs in particular highlight that operator education and a learning curve are some of the challenges in the clinical implementation of THV interventions in the Asia-Pacific area.^33^ Furthermore, the decrease in regurgitation from CoreValve to Evolut R and PRO is another example of the improvement in PVR with the introduction of an antileak skirt in the recent iterations of Evolut.

**Figure 2:**
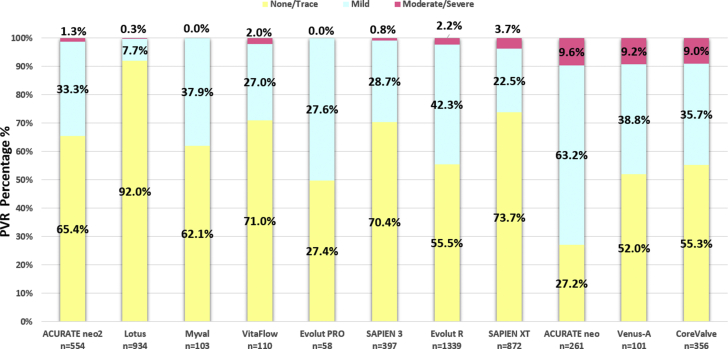
Paravalvular regurgitation (%) as reported by echocardiography (discharge or 30 ​days) in the main TAVR trials. TAVR, transcatheter aortic valve replacement.

Our findings show higher proportions of moderate/severe AR than those reported with echocardiography^1^^,^^32^^,^^34^, ^35^, ^36^, ^37^, ^38^, ^39^, ^40^, ^41^ (Figure 2), which is concordant with the results of the CHOICE trial,^22^ wherein the percentage of more than mild AR was 18.1% for self-expanding and 4.1% for balloon-expandable THVs by angiography in comparison to 5.8% and 1.6% by echocardiography, respectively. There are several possible reasons for these differences; first, the patient cohorts in these trials were selected according to detailed inclusion/exclusion criteria in contrast to our pooled analysis cohort, which was real-world all-comers. Second, paravalvular leak (PVL) assessment is traditionally detected and semiquantified by echocardiography; however, this modality of examination is not completely exempt from limitations, eg, assessment of AR is significantly influenced by the imaging plane, with low sensitivity in detecting posterior jets in the short-axis view,^42^ while significant variability also exists in the reported incidence of “what is mild” vs “what is moderate” among different core labs (Kappa ​= ​0.481 and 0.517 for PVL class 4 and PVL class 7 grading, respectively).^43^ Third, in our study, we assessed immediate regurgitation (within seconds to minutes of implantation), as opposed to echocardiographic assessment, which is usually performed at discharge or 30 days after TAVR.^37^^,^^44^ On the other hand, the sealing feature of the antileak skirts of novel devices may take minutes to achieve their complete functionality. Thus, deferred echocardiography may assess regurgitation with a fully operating antileak skirt, with a consequently lower amount of regurgitation. However, we reported a good correlation between the AR measured immediately after procedure using videodensitometric assessment and by CMR performed on average 10 ​days after procedure.^16^ In addition, in 2 cohorts of patients, we correlated the LVOT-AR assessed immediately after procedure with transthoracic echocardiography before discharge and showed that the categorical degree of regurgitation was concordant between the 2 methods.^16^ Moreover, data on the late reduction or increase of paravalvular leakage are inconsistent in the literature.^45^ In BEV THVs, recoil could be a potential factor for the late increase, whereas with SEVs, late self-expansion could explain the reduction in PVL. In addition to these opposing effects, the internal and external skirt (potentially absorbing fluid) may also affect the progression in PVL over time. Notwithstanding this, we should emphasize that when a PVL is moderate/severe based on the videodensitometric criteria of ≥17%, a direct corrective intervention should be performed considering its poor long-term prognosis.^15^ PVLs observed before discharge or at 30-day follow-up can only be amended by a paravalvular plug.

The rate of mild PVR published in different studies ranges from 5.9% to 63.4%.^46^ In our analysis, mild PVR ranges from 19% to 52% with no significant improvement in comparison with first-generation THVs. Mild PVR has been associated with poor outcomes in some^47^^,^^48^ but not all studies.^49^, ^50^, ^51^ Previous meta-analyses reported that mild PVR was associated with increased mortality.^46^^,^^52^ This discrepancy between different studies may be due to misclassification of the grade of AR. Indeed, significant variability in the reported incidence of “what is mild” vs “what is moderate” has been reported among different core labs.^43^ This debate requires further investigation to establish whether mild AR has a benign or malignant outcome and whether further refinement of THVs will be able to eliminate paravalvular and transvalvular regurgitation. Quantitative videodensitometric analysis of AR has clear well-validated cutoffs for grading AR, which could facilitate its role in helping overcome these challenges.

Lastly, the 4 added valves are not only new with different designs, but also represent more recent TAVR procedures. As such, there have been improvements in technique, use of computed tomography imaging for sizing, and expanded TAVR indications to lower-risk patients. Moreover, operator experience with upsloping learning curves for TAVR over time may have also influenced rates of PVR.

### Limitations

This was a pooled analysis of real-world patients undergoing TAVR; no randomization was performed for valve comparison, which may inherently lead to selection bias. Assessment of AR following TAVR with LVOT-AR is being utilized in ongoing randomized trials, eg, the LANDMARK trial^8^ comparing 2 BEVs and 1 SEV, and may confirm the accuracy of previous retrospective analyses. The percentage of aortograms that were suitable for videodensitometric analysis was around 60%, which is similar to previous retrospective series. The present analysis aimed to show the capability of videodensitometry in detecting different grades of regurgitation among THVs. Moving forward, this technique is only applicable to a randomized trial, eg, LANDMARK trial (NCT04275726) and Compare TAVR trial (NCT04443023), as well as in the ongoing registries evaluating the ACURATE neo2 (NCT04810195) with a standardized protocol of acquisition to increase the feasibility of analysis to 95%, as demonstrated in the ASSESS-REGURGE study^12^ and OVAL trial (Online Video-densitometric Assessment of Aortic Regurgitation in the Cath-Lab; NCT04047082).^6^ The purpose of the present analysis was to show the various grades of regurgitation among different valves, but no information regarding calcification, presence of bicuspid valves, aortic annular size and shape, THV diameter, technique, and depth of implantation was collected. Moreover, we only present data on their acute performance. One of the limitations of 2-dimensional videodensitometry is the lack of 3-dimensional localization of the regurgitation jet. Development of 3-dimensional videodensitometric analysis from biplane aortography is ongoing. Also, our pooled analysis did not include the recent iterations, Sapien 3 Ultra and Evolut PRO+.

## Conclusion

After videodensitometric analysis of 2665 aortographies, the Lotus valve had the lowest mean AR, while the ACURATE neo2 had the lowest percentage of moderate/severe AR, with significant improvements seen in comparison to ACURATE neo. Myval, VitaFlow, and Venus-A are promising options in the THV armamentarium. These results need to be confirmed in prospective randomized, head-to-head comparisons between THVs.

## Declaration of competing interest

Prof. Serruys reports personal fees from Biosensors, Medtronic, Micel Technologies, Sinomedical Sciences Technology, St. Jude Medical, Philips/Volcano, Xeltis, and HeartFlow, outside the submitted work. Dr Rück reports grants, personal fees, and nonfinancial support from Boston Scientific, nonfinancial support from Medtronic, and personal fees from Edwards, outside the submitted work. Dr Kim is a proctor/speaker/advisory board member to Abbott, Boston, Edwards, Medtronic, Meril, and Shockwave. Dr Mylotte is a consultant for Medtronic, Boston Scientific, and Microport. Prof. Soliman and Prof. Onuma report several institutional research grants outside the submitted work. All other authors have no conflict of interest to declare.
