# Supplementary material for: Quantitative Angiographic Assessment of Aortic Regurgitation Following 11 TAVR Devices: An Update of a Multicenter Pooled Analysis
Source: J Soc Cardiovasc Angiogr Interv. 2022 Apr 14;1(3):100037. doi: 10.1016/j.jscai.2022.100037 (PMC11308439; doi:10.1016/j.jscai.2022.100037)
Supplement: Supplemental Tables 1 and 2 [file mmc2.docx]

**Supplemental Table 1.** List of collaborating centers, investigators, recruitment period, and operator experience (of Modolo et al 2020 cohort).

| **Investigators** | **Centers** | **Recruitment period** | **Operator experience/valve stage** |
| --- | --- | --- | --- |
| Lars Sondergaard | The Heart Center, Rigshospitalet, University of Copenhagen, Denmark | Jan 2010 – Apr 2013 | Expert/routine |
| Pedro A. Lemos Fabio Sandoli Jr | Heart Institute (InCor), University of São Paulo Medical School, São Paulo, Brazil | Jan 2010 – Nov 2015 | Expert/routine |
| Jan-Malte Sinning Baravan Al-Kassou | Universitätsklinikum Bonn, Bonn, Germany | Jan 2010 – Oct 2018 | Expert/routine |
| Kyohei Yamaji | Kokura Memorial Hospital, Kitakyushu, Japan | May 2016 – Jun 2018 | Learning curve /routine |
| Nicolas M Van Mieghem | Erasmus Medical Center, Rotterdam, the Netherlands | Jan 2017 – Mar 2019 | Expert/routine |
| Hiroki Tateishi Yosuke Miyazaki | Yamaguchi University, Yamaguchi, Japan | Jan 2017 – Sep 2019 | Learning curve/routine |
| Alaide Chieffo | IRCCS San Raffaele Scientific Institute, Milan, Italy | Jun 2017 – Jul 2018 | Expert/routine |
| Nicolo Piazza Michele Pighi | McGill University Health Centre, Montreal, Canada | Jul 2017 – Jan 2019 | Expert/routine |
| Mohamed Abdel-Wahab Gert Richardt Mohammad Abdelghani | Segeberger Kliniken, Bad Segeberg, Germany | Aug 2017 – Mar 2018 | Expert/routine |
| Didier Tchetche | Clinique Pasteur. Toulouse, France | Aug 2017 – Jul 2018 | Expert/routine |
| Robbert de Winter Joanna J. Wykrzykowska | Amsterdam University Medical Center, Amsterdam, the Netherlands | Feb 2018 – Oct 2019 | Expert/routine |
| Andreas Ruck | Karolinska University Hospital, Stockholm, Sweden | Jul 2018 – Jun 2019 | Expert/early experience |

**Supplemental Table 2.** List of collaborating centers, investigators, recruitment period, and operator experience (of the four newly added THVs).

| **Valve** | **Investigators** | **Centers** | **Recruitment period** | **Operator experience/ valve stage** |
| --- | --- | --- | --- | --- |
| Venus A | Ling Tao and Jian Yang | Xijing Hospital, Xi’an, China | Mar 2016- Oct 2019 | Learning curve/First In Man |
| VitaFlow | Ling Tao and Jian Yang | Xijing hospital, Xi’an, China | Sep 2019 - May 2021 | Learning curve/ First In Man |
|  | Jianfang Luo | Guangdong Cardiovascular Institute, Guangdong Provincial People’s Hospital, Guangdong Academy of Medical Sciences, Guangzhou, China |  |  |
|  | Junjie Zhang | Nanjing First Hospital, Nanjing Medical University, Nanjing, China |  |  |
|  | Ping Li | Yulin First People’s Hospital, The Sixth Affiliated Hospital of Guangxi Medical University, Yulin, China |  |  |
| Myval  (Multicenter European registry of the Myval) | Dariusz Jagielak | Department of Cardiac and Vascular Surgery, Medical University of Gdansk, Gdansk, Poland | May 2019- Sep 2020 | Expert/early experience |
|  | Frederico De Marco | Clinical and Interventional Cardiology Department, IRCCS Policlinico San Donato, San Donato Milanese, Italy |  |  |
|  | Alfonso Ielasi | Interventional Cardiology Unit, Istituto Clinico Sant’Ambrogio, Milan, Italy |  |  |
|  | Peter den Heijer | Department of Cardiology, Amphia Ziekenhuis, Breda, the Netherland |  |  |
|  | Christian Juhl Terkelsen | Department of Cardiology, Aarhus University Hospital, Aarhus, Denmark |  |  |
| ACURATE neo2  (Early Neo2 Registry) | Andreas Rück  Christopher U. Meduri | Karolinska University Hospital, Stockholm, Sweden | Sep 2020- Jan 2021 | Expert/early experience |
|  | Won-Keun Kim | Kerckhoff Heart Center, Bad Nauheim, Germany |  |  |
